# Role of Cytokines and Toll-Like Receptors in the Immunopathogenesis of Guillain-Barré Syndrome

**DOI:** 10.1155/2014/758639

**Published:** 2014-09-22

**Authors:** Kishan Kumar Nyati, Kashi Nath Prasad

**Affiliations:** ^1^Laboratory of Immune Regulation, Immunology Frontier Research Center, Osaka University, Osaka 5650871, Japan; ^2^Department of Microbiology, Sanjay Gandhi Postgraduate Institute of Medical Sciences, Raebareli Road, Lucknow 226014, India

## Abstract

Guillain-Barré syndrome (GBS) is an autoimmune disease of the peripheral nervous system, mostly triggered by an aberrant immune response to an infectious pathogen. Although several infections have been implicated in the pathogenesis of GBS, not all such infected individuals develop this disease. Moreover, infection with a single agent might also lead to different subtypes of GBS emphasizing the role of host factors in the development of GBS. The host factors regulate a broad range of inflammatory processes that are involved in the pathogenesis of autoimmune diseases including GBS. Evidences suggest that systemically and locally released cytokines and their involvement in immune-mediated demyelination and axonal damage of peripheral nerves are important in the pathogenesis of GBS. Toll-like receptors (TLRs) link innate and adaptive immunity through transcription of several proinflammatory cytokines. TLR genes may increase susceptibility to microbial infections; an attenuated immune response towards antigen and downregulation of cytokines occurs due to mutation in the gene. Herein, we discuss the crucial role of host factors such as cytokines and TLRs that activate the immune response and are involved in the pathogenesis of the disease.

## 1. Introduction

Guillain-Barré syndrome (GBS), an immune-mediated polyneuropathy, is characterized by an autoreactive leukocyte infiltration into the peripheral nervous system (PNS) with neuroinflammation, demyelination, and axonal degeneration. The incidence of GBS ranges from 1 to 2 cases per 100,000 populations each year. Among the three major subtypes, acute inflammatory demyelinating polyneuropathy (AIDP) is the most common form of GBS in Europe and North America [1, 2]. In AIDP, the immune system reacts against target epitopes in Schwann cells or myelin resulting in demyelination. Experimental autoimmune neuritis (EAN), a T cell mediated disease in Lewis rats, is considered an animal model of AIDP [3] which is developed by injecting proteins and peptides derived from myelin of the PNS inducing similar pathologic features of AIDP. In brief, a bacterial protein epitope that is presented by a macrophage to T cell, which penetrates the endothelium, recognizes a cross-reactive antigen which results in releasing cytokines that activate endoneurial macrophages. These release enzymes and nitric oxide radical and ultimately invade compact myelin. In parallel, activated T cell releases cytokines, helps B cells to produce antibodies that cross damaged blood-nerve barrier (BNB), engages unidentified epitopes on abaxonal Schwann cell surface, fixes complement, damages Schwann cell, and produces vesicular dissolution of myelin. In contrast, acute motor axonal neuropathy (AMAN), an antibody-mediated disorder with little or no inflammatory infiltrates, occurs more frequently in East Asia mainly in China and Japan [2]. In the AMAN form of GBS, the infecting organisms probably share homologous epitopes to a component of the peripheral nerves and, therefore, the immune responses cross-react with the nerves causing axonal degeneration. The target molecules in AMAN are likely to be gangliosides GM1, GM1b, GD1a, and GalNAc-GD1a expressed on the motor axolemma. Rabbits have also been reported to develop a sensory and motor neuropathy following immunization with GD1a and GM1 or LOS extracted by* Campylobacter jejuni* (*C. jejuni*) containing ganglioside-like epitopes and the findings correspond well with pathological findings for human AMAN. Moreover, a bacterial ganglioside-like epitope stimulates B cells to induce antibodies that opsonize cross-reactive axolemmal antigens, fix complement and target macrophages to invade the periaxonal space, block conduction, or cause axonal degeneration.

Both humoral and cellular immune responses have been implicated in the pathogenesis of GBS and associated with autoantibodies and activated lymphocytes, respectively, which work in coordination in the pathogenesis of GBS. This review largely focuses on cellular immune response and associated host factors during the course of the disease. However, there are several evidences which show the involvement of humoral immunity in GBS such as the demonstration of immunoglobulin and complement deposition in GBS nerve biopsy specimens [4–7], the therapeutic effect of plasmapheresis and intravenous immunoglobulin (IVIg), the ability of GBS sera to cause demyelination of nerve, and the demonstration of antibodies against peripheral nerve constituents in GBS sera. Whole serum or IgM antibody from some patients with GBS produces complement-dependent demyelination of peripheral nerve [8–10]. In GBS, activated complement components are detectable in serum and cerebrospinal fluid (CSF). Anti-myelin antibodies peak prior to activated complement levels, providing evidence of an antibody-mediated complement attack on peripheral nerve myelin [11, 12]. The extent of nerve damage depends on several factors which leads to weakness and may cause conduction disturbances.

Several antecedent infections such as* C. jejuni*,* Cytomegalovirus*, Epstein-Barr virus, and* Mycoplasma pneumoniae* have been detected in GBS patients but their role as triggering agent except* C. jejuni* remains inconclusive. The absolute mechanisms involved in pathogenesis of GBS are still unclear; however, the hypothesis put forward for the immunopathogenesis of GBS is the molecular mimicry between lipopolysaccharides (LPS) and ganglioside-like epitopes in host nerve cells, which leads to cross-reactivity of immune response following infection. However, not every individual infected by the above infectious agents develops GBS. Less than 1* C. jejuni* infected individual in 1000 secrete antibodies that bind the cross-reactive epitopes and cause the paralyzing GBS [13]. Several observations draw attention to the significance of the host factors in the pathogenesis of GBS such as several* C. jejuni* strains that have GM1 ganglioside-like epitopes but they fail to induce anti-ganglioside antibodies. Despite the molecular mimicry by* C. jejuni* LPS, some people develop only a particular form of GBS. This phenomenon strongly suggests the involvement of some other factors in the development of GBS after infection. There may be disease susceptibility genes that may predispose certain individuals to develop GBS after being infected with different microbial agent. Moreover, host factors determine the immune response towards LPS which can play crucial role in the pathogenesis of the disease and its differential manifestations in different areas of the world. However, studies are elusive in identifying potential host factors involved in the disease pathogenesis and impart susceptibility to an individual for the development of GBS.

In this regard, cytokines and toll-like receptors (TLRs) can play important role as they are involved in many inflammatory and autoimmune diseases by activating the immune response towards pathogens via initiating cascade for cytokine and chemokine production. TLRs comprise a family of structurally related receptors that recognize specific parts of microorganisms and endogenous ligands associated with cell damage. They have a capacity to directly recognize diverse pathogen-associated molecular patterns (PAMPs) that are unique to microorganism and therefore absent from host cells. This makes them well suited to act as an early warning system against invading pathogens and leads to increase in expression of proinflammatory cytokines such as IL1, IL6, and TNF*α* which can help in T cell differentiation and further directing the host immune response [14, 15]. In the present review, we focus on the role of the cytokines and TLRs as promoters and mediators of cross-reactions initiating innate and adaptive immune response that help to generate molecular mimicry triggering the inflammatory response in GBS.

## 2. Cytokines and GBS

**Figure 1:**
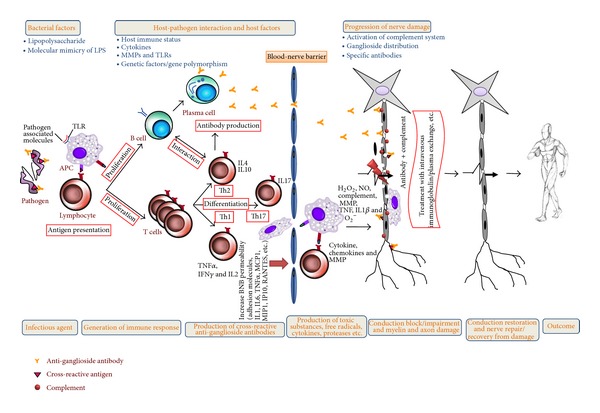
Overview of immunopathogenesis of GBS. A bacterial cross-reactive antigen recognized by macrophages and T cells that help B cells to produce anti-ganglioside antibodies which penetrates blood-nerve barrier and can activate complement. These antibodies bind with specific nerve gangliosides and antigen as well. Activated endoneurial macrophages release cytokines, proteases, and free radicals (nitric oxide, oxygen, and hydrogen peroxide), invade compact myelin and periaxonal space, and sometimes block nerve conduction or cause axonal degeneration. Activated T cell releases proinflammatory cytokines, fixes complement, damages Schwann cell, and ultimately produces dissolution of myelin. The extent of nerve damage depends on several factors. Nerve dysfunction leads to weakness and might cause sensory disturbances. Treatment with IVIg and/or PE helps in recovery from the disease; however despite IVIg/PE treatment, many patients only partially recover and have residual weakness, pain, and fatigue. BNB, blood-nerve barrier; TNF*α*, tumor necrosis factor alpha; IL, interleukin; IFN*γ*, interferon gamma; APC, antigen presenting cell; TLR, toll-like receptor; Th, helper T cell; IVIg, intravenous immunoglobulin; PE, plasma exchange.

Cytokines are small proteins that are involved in the process of inflammation, including the initiation and amplification of leukocyte recruitment into the PNS. They act by regulating cellular replication, differentiation, or activation. Th1 cytokines (IFN*γ* and IL12) are believed to play an important role in the induction of cell-mediated autoimmune disease whereas Th2 cytokines (IL4) promote primarily antibody-mediated autoimmune disease. There are many reports which suggest that cytokines are significantly involved in immune-mediated demyelination of peripheral nerves. In case of experimental autoimmune encephalitis (EAE), an animal model for immune-mediated inflammation of central nervous system, there are studies which suggest that there is differential upregulation and expression of various cytokines by infiltrating lymphocytes and residential cells. Studies also suggest the role of cytokines in the pathogenesis of EAN. In GBS, cytokines can be produced in the peripheral nerves by infiltrating mononuclear cells and Schwann cells in AIDP and by macrophages in the axonal forms of GBS. It is suggested that a cascade of immune-mediated inflammatory responses can be generated by specific immune recognition involving T lymphocytes, monocytes, and various cytokines responsible for causing demyelination in the host PNS. These cytokines may assist in the disruption of the BNB; as a result of which, immune cells can infiltrate through the barrier and directly access myelin and Schwann cells, thus affecting the peripheral nerve conduction (Figure 1).

Studies are being carried out to know the exact role of cytokines in GBS and cascade of events leading to demyelination and axonal damage, particularly TNF*α*. It may act in the acute or afferent phase of an immune reaction and likely contributes to the development of inflammatory and immunopathologic lesions in many autoimmune diseases including GBS. In GBS, TNF*α* produced by the infiltrating T cells has a direct myelinotoxic effect on myelinated fibers, causing demyelination. Moreover, it can affect the synthesis of myelin protein and glycolipids [16]. As a typical marker for a Th1 response, IFN*γ*, produced by Th1 cells, exerts its proinflammatory role by activating endothelial cells, macrophages, and T cells. IFN*γ* increases the expression of major histocompatibility complex II thereby enhancing the antigen presenting capacity of macrophages. The potent proinflammatory activities of IFN*γ* combined with its inhibitory potential on Th2 cells make IFN*γ* a central mediator of Th1 mediated autoimmune disorders. In addition, IFN*γ* induces the differentiation of T cells to a Th1 phenotype, B cell class switching, apoptosis of T cell, and enhancement of production of other cytokines such as TNF*α*, IL1*β*, and IL6. IL1 has been shown to participate in the repair and regeneration of PNS and as GBS is a self-limiting disorder, the role of IL1 can be of interest during the recovery phase of GBS. Increased IL10 expression in the early phase of GBS downregulates Th1 cytokine synthesis and may act as a physiologic countermeasure of immunologic mediators of neuroinflammation [17, 18]; however, the role of IL10 remains contradictory.

Excretion of* C. jejuni* usually ends almost in 16 days after onset of diarrhea [19] and the GBS typically occurs 3-4 weeks after diarrhea [20]. When GBS establishes, in most of the cases,* C. jejuni* infection may have cleared but the immune response generated following infection continues during the course of illness. To diagnose* C. jejuni* infection in such GBS cases, we have previously evaluated the lymphocyte transformation test (LTT), a test that measures the proliferation of T cells to an antigen in vitro to identify a previous in vivo reaction due to a sensitization, for the detection of a response to* C. jejuni* antigen in the lymphocytes from GBS patients [19]. In this study, we found that cell proliferation in GBS cases was significantly higher than the controls (*P* < 0.001) and test showed 77.5% sensitivity and 96.5% specificity which illustrates that activated lymphocytes might play role in the pathogenesis of neuronal damage in GBS. Subsequently, we have demonstrated a role of proinflammatory (IFN*γ*, IL1*β*, TNF*α*, and IL6) and anti-inflammatory (IL4, IL10, and TGF*β*) cytokines, in our GBS cohort [17] and in an experimental animal model [20], and concluded that Th1 cytokines in the early disease course were associated with immune-mediated disease progression due to neuronal inflammation, but Th2 immune response during the later phase helped in recovery from the disease. Studies in EAN showed a similar but unidentical trend [21]. Rats with EAN have increased levels of IFN*γ*, IL1*β*, TNF, and IL6 in the acute phase and increased IL4, TGF*β*, and IL10 levels in the recovery stage. In contrast to EAN, the immune-modulating cytokine IL10 was upregulated during the progressive phase of GBS as reported in our previous study [17].

Another cytokine IL23 is an important part of the inflammatory response against infection. Knockout mice deficient in either p40/p19 or subunit of the IL23 receptor (IL23R and IL12R-*β*1) develop less severe symptoms of multiple sclerosis and inflammatory bowel disease highlighting the importance of IL23 in the inflammatory pathway [22, 23]. Earlier it has been suggested that IL23 may play an important role during the early effector phase in immune-mediated demyelination of the peripheral nerve [24]. In conjunction with IL6 and TGF*β*1, IL23 stimulates naïve CD4+ T cells to differentiate into a novel subset of cells called Th17 cells. These cells produce IL17 that stimulates the production of proinflammatory cytokines such as IL1*β* and IL6 from monocytes and therefore further amplifies the inflammatory cascade [25]. IL17 producing Th17 cells are associated with immunopathology in autoimmune diseases. Recently, the role of Th17 cells has been shown and correlated with the pathogenesis of GBS. IL17, a signature cytokine produce by Th17 cells, may have synergistic effects with proinflammatory cytokines such as TNF*α*, IFN*γ*, and IL1*β*. IFN*γ* can prevent IL23 triggered expansion of Th17 cells [26]; therefore, IFN*γ* sometimes plays a protective role in GBS/EAN which might be due to its ability to suppress Th17 development. Moreover, IFN*γ* increases T-bet expression, the overexpression of which in turn leads to a robust reduction of IL17 generation [26, 27]. Further, IL17 was detected in sciatic nerves of EAN, and the accumulation of IL17 was correlated with the severity of neurological signs [28], which suggests a pathological contribution of IL17 to the development of EAN. The frequency of Th17 cells in CSF and the level of IL17 in plasma were significantly higher in active chronic inflammatory demyelinating polyradiculoneuropathy (CIDP) [29]. Further the levels of IL17 and IL22 in CSF were correlated with GBS severity [30]. Liang et al. [31] suggested that the TIM-3 pathway influenced IL17 release and Th17 and Th1 differentiation along with their cytokine expressions during the pathogenesis of GBS. Pelidou et al. [32] reported enhanced acute phase of EAN in Lewis rats by intranasal administration of recombinant mouse IL17, along with increased infiltration of inflammatory cells into the sciatic nerves and severe demyelination. Collectively, these findings indicate that Th17 cells and their effector cytokines might be involved in the pathogenesis of GBS and EAN. Although the mechanism of action of IL17 in GBS and EAN remains unclear, it mainly acts as a proinflammatory cytokine that upregulates the expression of inflammatory genes including proinflammatory chemokines, hematopoietic cytokines, acute phase response genes, and antimicrobial substances [33] in neutrophils, macrophages, and endothelial cells [34].

IL37, a member of the IL1 cytokine family, has been thought to be an anti-inflammatory cytokine produced by several types of cells. A recent study suggests that proinflammatory cytokines may promote anti-inflammatory IL37 expression to downregulate excessive inflammation during the pathogenic process of GBS [35]. Indeed, IL37 has been shown to inhibit proinflammatory responses in mice [36, 37]. Interestingly, IL17A can disrupt the BNB [38], and the concentrations of CSF IL17A and IL37 were correlated positively in GBS patients. It is also possible that IL17A might drive the entry of IL37 from plasma to the CNS in GBS patients and the levels of plasma and CSF IL37 might be useful for the evaluation of disease severity in GBS patients [35].

## 3. Toll-Like Receptors and GBS

**Figure 2:**
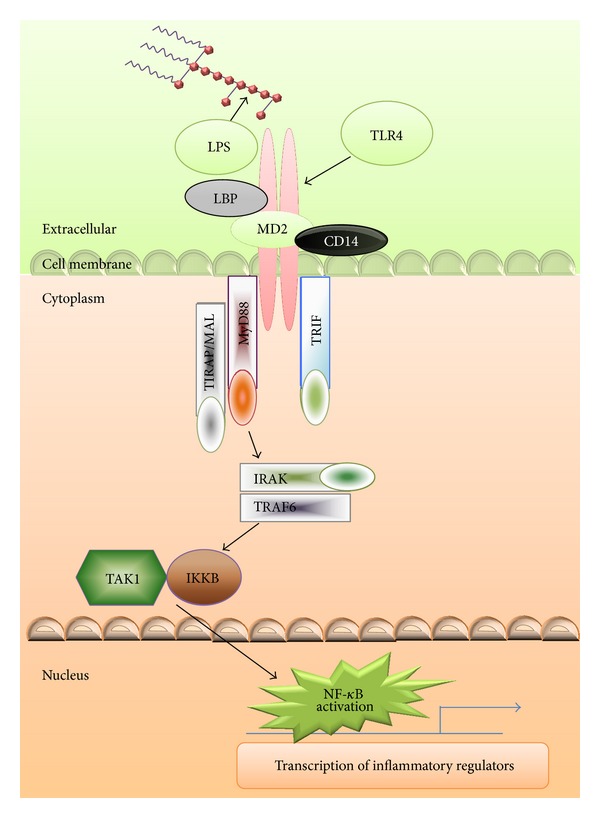
TLR4 signaling during the host-pathogen interaction: pattern recognition receptors (PRRs) recognize evolutionary conserved repetitive structures such as lipid A in LPS present in* Campylobacter jejuni* and various other microorganisms. Stimulation of TLR4 by LPS facilitates the activation of two pathways: the MyD88- (myeloid differentiation primary-response protein 88-) dependent and MyD88-independent pathways (not shown). Downstream of TLR4 signaling involves different types of adaptor molecules which depend on the type of LPS and result in early phase of NF-*κ*B activation, which leads to the production of inflammatory cytokines. LPS, lipopolysaccharide; LBP, lipopolysaccharide binding protein; TLR4, toll-like receptor 4; MAL, MyD88 adaptor-like; TRIF, TIR domain-containing adaptor-inducing IFN-*β*; TRAF, TNF receptor-associated factors; TIRAP, toll-interleukin 1 receptor (TIR) domain-containing adaptor protein; IRAK, IL-1 receptor-associated kinase; TAK, TGF*β*-activated kinase; IKKB, I*κ*B kinases; NF-*κ*B, nuclear factor kappa-light-chain-enhancer of activated B cells.

TLRs are mainly transmembrane proteins and are members of the pattern recognition receptors (PRRs) family. They play a central role in the initiation of both innate and adaptive immune responses against microbial pathogens through MyD88- (myeloid differentiation primary-response gene-) dependent or MyD88-independent transduction pathway [39]. Each member of the TLR family has its own ligand from different pathogen, which helps in inducing a danger signal when pathogen invades the host and results in the activation of NF-*κ*B and subsequent induction of signal transduction cascade (Figure 2). TLR signaling pathways are essential for protection against diseases, but there are many studies which show that excessive signaling may lead to allergies, atherosclerosis, and autoimmune diseases. In case of autoimmune diseases, the TLR signaling leads to activation of self-reactive T or B cells. The activation of such self-reactive cells may be due to the presence of some danger signals, derived from microorganisms which break immunological tolerance of the host, and further leads to development of autoimmune disease. Therefore, these molecules can act as crucial mediators for detecting these danger signals and inducing the signaling pathways related to host defense.

Meanwhile, TLRs can also activate the antigen presenting cells (APCs) such as dendritic cells (DCs) through MyD88-dependent or MyD88-independent pathway to start the adaptive immunity. More recently, the first systematic analysis of TLR expression pattern has suggested the role of TLRs in GBS pathophysiology [40]. In GBS, ganglioside mimicry of* C. jejuni* lipooligosaccharide (LOS) drives the production of cross-reactive antibodies to peripheral nerve gangliosides. However, the mechanism for this aberrant humoral immune response to* C. jejuni* in GBS is unknown. It was reported that human DC activation and subsequent B-cell proliferation are modulated by sialic acid residues in GBS-associated* C. jejuni* LOS. Sialylated LOS of* C. jejuni* isolates, strongly associated with the development of GBS, induced human DC maturation and secretion of inflammatory cytokines that were mediated by TLR4. The TLR4/MD2 receptor complex could have a higher affinity for the LOS-LBP-CD14 complex when LOS is sialylated. The extent of TLR4 signaling and DC activation was found higher with sialylated-LOS, indicating that sialylation boosts the DC response to* C. jejuni* LOS and may contribute to the development of cross-reactive anti-ganglioside antibodies found in GBS patients following* C. jejuni* infection [41, 42]. TLR2 can deliver costimulatory T cell signals for cell expansion and can induce proliferation of regulatory T cells [43]; its signaling favors Th17 cell expansion. It is shown in the rat model of EAN that TLR2 is expressed in inflamed nervous tissue [44] and NF*κ*B is increased in activated T cells and macrophages. Even Schwann cells subsequently modulate the expression of proinflammatory cytokines, chemokines, or enzymes such as IL1*β*, TNF*α*, and iNOS [45]. Furthermore, in EAN, potential endogenous TLR ligands generated following tissue damage or inflammation may also activate their TLRs and thereby play roles in the pathological progress of the disease. TLR2+, CD14+, and Hsp70+cell accumulation was detected and positively correlated with neurologic disease severity in sciatic nerves of EAN rats, suggesting the involvement of innate immunity in the effector phase of disease. This study also suggested that upregulated Hsp70 might function as an endogenous ligand of TLR2 to induce expression of cytokines, like IL12, to contribute to the progress of EAN [44]. Studies showed that TLR2 and TLR6 expression were significantly elevated in lymph node cells and sciatic nerves during EAN and GBS [44, 46]. EAN induced CD4+ T cells showed a highly significant increase in both TLR2 and TLR6 expression at 6 days of postimmunization (dpi) and at 13 dpi. CD8+ T cells showed an increase of TLR2 at the peak of disease (at 20 dpi), while TLR6 was already strongly increased in the early clinical stage (at 13 dpi). Major histocompatibility complex (MHCII+) APCs express markedly increased TLR2 levels during disease induction phase (at 6 dpi) and TLR6 was significantly elevated in the early clinical stage of the disease (at 13 dpi). Amongst others, TLR2 signaling requires heterodimerization with TLR6 [47], hinting towards a regulative role of TLR6, as it increases TLR2, which delivers potent costimulatory signals to antigen-activated T cells, and influences T cell proliferation, survival, and effector functions [48]. Moreover, it has been reported that TLR2/TLR6 heterodimers are able to detect diacylated thioester linkages to cysteinyl residues. Therefore, it may be of importance for EAN induction as the neuritogenic and immunogenic features of P0 are caused by its diacylation [49]. TLR4 and TLR9 recognize LPS of Gram-negative bacteria and unmethylated CpG DNA or some viruses, respectively. Studies have proved that TLR4 and TLR9 were upregulated during EAN and showed the role of TLR4 and TLR9 in the pathogenesis of EAN [36, 50]. TLR4 can induce Th17 differentiation and TLR8 can reverse the suppressive function of regulatory T cells. The TLR adaptor molecule, MyD88, plays a pivotal role in the development of EAE [51]. CD4+ and CD8+ T cells showed a significant increase of MyD88 expression throughout the induction phase and early clinical stage of the disease, while MHCII+ cells upregulation was only present in the early clinical stage. IL-17A seemed to play a role, particularly in the induction phase on CD4+ and CD8+ T cells, as the significant increase was not found after clinical manifestation. However, it needs attention and calls for the further study to find out a promising interaction between Th17 and TLRs in GBS.

Severity of clinical symptoms in EAN negatively correlated with the levels of TLR2, TLR6, and TLR4 while upregulation of TLR11 and downregulation of TLR1 were observed during the active phase of the disease. Upregulation of TLR11 was seen in CD4+, CD8+, and MHCII+ cells up to 13 dpi, only at 13 dpi and at 6 dpi, respectively. Decreased TLR1 expression was seen in CD4+ and CD8+ T cells at 2 dpi while in MHCII+ cells it was seen until 6 dpi. It has been reported that TLR11, expressed on dendritic cells, is crucial to prime CD4+ T cells during infection [52]. It has been recently reported that CD4+ T cells can directly respond to* Mycobacterium tuberculosis* products, which are used for immunization by forming TLR2/TLR1 heterodimer [53]. However, the functional role and exact mechanism of upregulation/downregulation of TLR11/TLR1 during this demyelinating disease still require further research.

## 4. Cytokine- and TLR-Gene Polymorphisms and GBS

As discussed earlier, epidemiological studies reported that about one in 1000* C. jejuni* enteritis patients developed GBS. The* C. jejuni* genes responsible for the development of GBS alone do not sufficiently explain the reason behind the autoimmune response being triggered only in a minority of individuals with* C. jejuni* enteritis. Occurrence of GBS within families suggests that host susceptibility is also important [54]. Previous attempts to find common host immunogenetic factors among* C. jejuni*-related GBS patients, however, have been negative or diverse for HLA typing [13, 55, 56], T cell receptor genotyping [57], and polymorphism analysis of CD14 and TLR4 [54]. Since many genetic polymorphisms have been identified in the regulatory or coding regions of cytokines and other inflammatory mediators that may affect their expression and function, it is plausible that genetic polymorphisms in inflammatory genes may predict susceptibility and risk for development of GBS, thus proving to be potential markers for the disease.

TNF*α* is a good candidate gene for the study of autoimmune disease because it codes for important immunoregulatory cytokines. Recently, we have also investigated TNF*α* polymorphisms (−308 G/A, −863 C/A, and −857 C/T) and their expression in GBS patients and found that −308 G/A and −857 C/T polymorphisms with increased TNF*α* levels may predict susceptibility to axonal subtypes of GBS [58]. IL10 is a cytokine that displays pleiotropic effects in immunoregulation and inflammation. IL10 inhibits Th1 production of IFN*γ* and IL2 and may have both pro- and anti-inflammatory effects in GBS. An SNP in the promoter region of IL10 associated with high IL10 production was a susceptibility factor for the onset of GBS [59]. Most of the studies have concluded that recognition of LPS by Gram-negative bacteria, NF-*κ*B activation (Figure 2), and TNF*α* secretion are decreased due to mutations in TLR4 [60, 61]. During infectious condition, these variations lead to hyporesponsiveness towards LPS, reduced epithelial TLR4 density, and inflammatory cytokine response [62]. Very few studies have been reported on GBS and TLR polymorphism till date [54, 63]. Previously, we found a significant association of Asp299Gly TLR4 polymorphism in GBS patients [64]. TLR9 (located on chromosome 3p21.3) is potentially associated with autoimmune diseases, because it participates in the production of proinflammatory cytokines and the maturation of dendritic cells. Earlier, Deng and Zhou [50] found that TLR4 and TLR9 were upregulated during the disease course of EAN and have reported the role of these TLRs in the pathogenesis of the disease. Several other gene polymorphisms such as HLA B54 [55], HLA-Cw1 [55], HLA class II [56], CD1 [65], MBL2 [66], HLA-DRB1*0701 [67], Fas/CD95 [56], Fc*γ*R2A [67], Fc*γ*R3A [68], Fc*γ*R3B [68], SH2D2A [69], immunoglobulin KM gene [70], GR haplotypes [71], and MMP9 [72] are also studied and associated with the development or severity of GBS.

## 5. Therapeutic Utilities

The discovery that endogenous ligands as well as microbial components are recognized by TLRs and that small-molecular-mass synthetic compounds activate TLRs raised interest in these receptors as potential targets for the development of new therapies for various autoimmune diseases. However, very few studies reported the role of TLRs in GBS and none of them has showed TLRs as a therapeutic target. Treatment of GBS involves management of severely paralyzed patients with intensive care and ventilatory support and specific immunomodulating therapies that reduce the progressive phase of GBS. High-dose IVIg and plasma exchange aid in more rapid resolution of the disease. IVIg is suggested as an effective treatment for GBS [73]. The most frequently used IVIg regime is 0.4 g/kg/day for 5 days [73, 74]. The precise mechanisms by which IVIg exerts its action appear to be a combined effect of complement inactivation, neutralization of idiotypic antibodies, cytokine inhibition, and saturation of Fc receptors on macrophages. The observed long-term immunomodulatory effect of IVIg may be due to interference with the amplification phase of the immune response, which involves the proliferation of T lymphocytes. Our previous study with earlier published data suggested that IVIgs used for the treatment of GBS suppress the levels of proinflammatory cytokines such as TNF-*α* and IL-1*β* during recovery, but remained relatively high in untreated patients [17, 75]. Several comparative studies suggested superiority of IVIg over plasma exchange [76] because of its convenience, simplicity, and greater comfort for the patient. However, back pain meningeal reaction, fever, tachycardia, and headache during or within course of completing the infusion are known side effects of IVIg [77].

Plasma exchange removes potentially pathogenic molecules from the circulation such as antibodies, complement, cytokines, and inflammatory mediators [78]. Plasma exchange may also indirectly influence cellular immune response. Experimental data also suggests that, on treatment with plasma exchange, there is an increase in immunoglobulins and deviation in cytokine pattern, which decreases the efficacy of this treatment. Moreover, beyond the removal of immunoglobulins, plasma exchange may have an immunomodulatory effect on T cell shifting its Th1/Th2 balance towards Th2. Plasma exchange is beneficial within 4 weeks of symptom onset and the benefits would be greatest when treatment is given early [79]. The usual regime is to exchange 4–6 plasma volumes over 2 weeks [74, 79]. However, plasma exchange did not affect the percentage of patients with severe motor disability. Around 25% of the cases have reported relapse approximately 1-2 weeks after plasma exchange, which is supposed to result from antibody rebound and increased levels of peripheral myelin-directed antibodies [80]. The main limitations for use of plasma exchange would be availability of the technical expertise and support.

In contrast to plasma exchange and IVIg, corticosteroids are mainly ineffective in GBS [81] either alone or in combination with IVIg [82]. Several studies have failed to demonstrate improvement in disability after 4 weeks of treatment with steroids [83, 84]. There were reports that showed slight improvement in GBS patients treated with oral steroids compared to controls [83, 84]. Overall, corticosteroids are not recommended for the management of GBS.

## 6. Summary and Conclusion

GBS is a heterogeneous disorder with variable clinical and pathologic features reflecting different mechanisms to nerve tissue injury. Inflammation, demyelination, and axon degeneration are the major pathologic mechanisms that cause the clinical manifestations. The most widely accepted theory is that GBS is mediated by molecular mimicry between LPS on the cell envelope of* C. jejuni* and ganglioside epitopes on the human peripheral nerves that generates cross-reactive immune response, resulting in autoimmune-driven nerve damage. In spite of common* C. jejuni* infection in population, the frequency of developing GBS is quite low (1 : 1000). This illustrates the important role of host factors in addition to molecular mimicry in the production of cross-reactive antibodies leading GBS (Figure 1). Various SNPs have been studied in relation to GBS susceptibility, production of cross-reactive antibodies, severity of GBS, and outcome of GBS. Although promising results have been reported in these studies, finding a general host-genetic factor is difficult because of the many possible simultaneously active pathways, heterogeneity of the disease, and unknown interactions between pathogen and host.

Cytokines in GBS have been extensively studied and at times the conflicting results make it more complicated. Besides the complexity of the cytokines, this is partly due to the different methods used and aspects of cytokines observed in the animal model and clinical studies. Blocking agents of T cells across the BNB and agents that antagonize T cell cytokine-induced priming of macrophages may prove efficacious. Studies suggested that GBS follows a simplified model in which inflammatory cytokines (IFN*γ*, TNF*α*, IL6, and IL1) are disease promoting, while anti-inflammatory cytokines serve as a countermeasure to limit and modulate the inflammatory response; however, the exact mechanism remains elusive. Furthermore, the reality may be more complex because they might exert their dual roles through different signaling pathways in various conditions. Therefore, therapies on cytokines especially by regulating their pathways are important and promising. Moreover, Th17 and some cytokines such as IL17, IL21, IL27, IL35, and IL37 are recognized well in autoimmune diseases, though their possible roles as therapeutic target in GBS need to be further explored.

TLRs, innate immune receptors, can recognize the conserved motif on pathogens by pathogen-associated molecular patterns and promote the innate immune defense through different signaling pathway; TLRs signaling also promotes activation and maturation of antigen-specific APCs to start adaptive immunity. TLR2, 4, and 9 as well as their related signaling molecules are reported to have strong positive correlation with disease severity in GBS. Macrophage migration inhibitory factor may participate in the pathogenesis of GBS by modulating the LOS-induced response through TLR4 signaling pathway. Human DCs also express sialic acid binding Ig-like lectins that bind to* C. jejuni*, which may play a critical role in the efficiency of TLR4 signaling after stimulation with* C. jejuni* with sialylated LOS. TLR4 was shown upregulated on MHCII+ cells in EAN and GBS. The treatment of CpG oligodeoxynucleotides (ODN), a suppressive ODN, altered the expression of TLR9 in EAN, and this stimulus or inhibition is positively related to the clinical signs of EAN suggesting that TLR9 is related to the pathogenesis of EAN. TLR2/6 was reported significantly higher on T cells and APCs, in sciatic nerve infiltrates of EAN mice, and in blood of GBS patients whereas TLR1 was significantly downregulated in the induction phase on T cells and APCs. Furthermore, TLR11 expression was found augmented on CD4+ T cells during EAN progression. However, a thorough comprehensive study of TLRs with their definite molecular mechanism in GBS is still challenging.

At last, researchers and scientists should focus their research for unveiling the GBS pathogenesis dealing with interactions between pathogen and host and will need to work in a collaborative effort to dissect out following research field based on cascade of events during GBS pathogenesis: (1) types of infection, (2) identification of environmental and molecular risk factors, (3) aberrant coordination of immune response between host and pathogen during the progression of the disease, (4) immunological and molecular pathways, (5) antibody specificity and clinical correlation, and (6) involvement of host factors. With regard to* C. jejuni*-infected GBS patients, SNPs in genes of other molecules involved in LPS responses may be involved in conferring the susceptibility and clinical pattern of GBS. Clearly, we have only just begun to understand the outline of the host factors in GBS, and further investigation will likely provide new insights into the detailed interplay with the host during steady-state and disease processes.

## References

[1] Griffin JW, Li CY, Ho TW (1996). Pathology of the motor-sensory axonal guillain-barré syndrome. *Annals of Neurology*.

[2] Kuwabara S, Ogawara K, Misawa S (2004). Does Campylobacter jejuni infection elicit “demyelinating” Guillain-Barré syndrome. *Neurology*.

[3] Vriesendorp FJ (1997). Insights into *Campylobacter jejuni*-induced Guillain-Barre syndrome from the Lewis rat model of experimental allergic neuritis. *Journal of Infectious Diseases*.

[4] Hughes R, Atkinson P, Coates P, Hall S, Leibowitz S (1992). Sural nerve biopsies in Guillain-Barre syndrome: axonal degeneration and macrophage-associated demyelination and absence of cytomegalovirus genome. *Muscle and Nerve*.

[5] Brechenmacher C, Vital C, Deminiere C (1987). Guillain-Barre syndrome: an ultrastructural study of peripheral nerve in 65 patients. *Clinical Neuropathology*.

[6] Vallat JM, Leboutet MJ, Jauberteau MO, Tabaraud F, Couratier P, Akani F (1994). Widenings of the myelin lamellae in a typical Guillain-Barre syndrome. *Muscle and Nerve*.

[7] Griffin JW, Stoll G, Li CY, Tyor W, Cornblath DR (1990). Macrophage responses in inflammatory demyelinating neuropathies. *Annals of Neurology*.

[8] Sawant-Mane S, Clark MB, Koski CL (1991). In vitro demyelination by serum antibody from patients with Guillain-Barré syndrome requires terminal complement complexes. *Annals of Neurology*.

[9] Sawant-Mane S, Estep A, Koski CL (1994). Antibody of patients with Guillain-Barré syndrome mediates complement-dependent cytolysis of rat Schwann cells: susceptibility to cytolysis reflects Schwann cell phenotype. *Journal of Neuroimmunology*.

[10] Birchem R, Mithen FA, L'Empereur KM, Wessels MM (1987). Ultrastructural effects of Guillain-Barre serum in cultures containing only rat Schwann cells and dorsal root ganglion neurons. *Brain Research*.

[11] Hartung H-P, Schwenke C, Bitter-Suermann D, Toyka KV (1987). Guillain-barré syndrome: activated complement components C3a and C5a in CSF. *Neurology*.

[12] Koski CL, Sanders ME, Swoveland PT (1987). Activation of terminal components of complement in patients with Guillain-Barre syndrome and other demyelinating neuropathies. *Journal of Clinical Investigation*.

[13] Magira EE, Papaioakim M, Nachamkin I (2003). Differential distribution of HLA-DQ*β*/DR*β* epitopes in the two forms of Guillain-Barré syndrome, acute motor axonal neuropathy and acute inflammatory demyelinating polyneuropathy (AIDP): identification of DQ*β* epitopes associated with susceptibility to and protection from AIDP. *Journal of Immunology*.

[14] Akira S, Takeda K, Kaisho T (2001). Toll-like receptors: critical proteins linking innate and acquired immunity. *Nature Immunology*.

[15] Kopp EB, Medzhitov R (1999). The Toll-receptor family and control of innate immunity. *Current Opinion in Immunology*.

[16] Lisak RP, Skundric D, Bealmear B, Ragheb S (1997). The role of cytokines in Schwann cell damage, protection, and repair. *Journal of Infectious Diseases*.

[17] Nyati KK, Prasad KN, Rizwan A, Verma A, Paliwal VK (2011). TH1 and TH2 response to Campylobacter jejuni antigen in Guillain-Barré syndrome. *Archives of Neurology*.

[18] Press R, Deretzi G, Zou LP (2001). IL-10 and IFN-*γ* in Guillain-Barre syndrome. *Journal of Neuroimmunology*.

[19] Nyati KK, Prasad KN, Rizwan A, Verma A, Paliwal VK, Pradhan S (2010). Lymphocyte transformation test detects a response to *Campylobacter jejuni* antigens in patients with Guillain-Barré syndrome. *Medical Microbiology and Immunology*.

[20] Nyati KK, Prasad KN, Kharwar NK (2012). Immunopathology and Th1/Th2 immune response of *Campylobacter jejuni*-induced paralysis resembling Guillain-Barré syndrome in chicken. *Medical Microbiology and Immunology*.

[21] Zhu J, Mix E, Link H (1998). Cytokine production and the pathogenesis of experimental autoimmune neuritis and Gulllain-Barre syndrome. *Journal of Neuroimmunology*.

[22] Kikly K, Liu L, Na S, Sedgwick JD (2006). The IL-23/Th17 axis: therapeutic targets for autoimmune inflammation. *Current Opinion in Immunology*.

[23] Langowski JL, Zhang X, Wu L (2006). IL-23 promotes tumour incidence and growth. *Nature*.

[24] Hu W, Dehmel T, Pirhonen J, Hartung H-P, Kieseier BC (2006). Interleukin 23 in acute inflammatory demyelination of the peripheral nerve. *Archives of Neurology*.

[25] Chabaud M, Aarvak T, Garnero P, Natvig JB, Miossec P (2001). Potential contribution of IL-17-producing Th1 cells to defective repair activity in joint inflammation: partial correction with Th2-promoting conditions. *Cytokine*.

[26] Harrington LE, Hatton RD, Mangan PR (2005). Interleukin 17-producing CD4^+^ effector T cells develop via a lineage distinct from the T helper type 1 and 2 lineages. *Nature Immunology*.

[27] Mathur AN, Chang H-C, Zisoulis DG (2006). T-bet is a critical determinant in the instability of the IL-17-secreting T-helper phenotype. *Blood*.

[28] Zhang Z, Zhang Z-Y, Schluesener HJ (2009). Compound A, a plant origin ligand of glucocorticoid receptors, increases regulatory T cells and M2 macrophages to attenuate experimental autoimmune neuritis with reduced side effects. *Journal of Immunology*.

[29] Chi LJ, Xu WH, Zhang ZW, Huang HT, Zhang LM, Zhou J (2010). Distribution of Th17 cells and Th1 cells in peripheral blood and cerebrospinal fluid in chronic inflammatory demyelinating polyradiculoneuropathy. *Journal of the Peripheral Nervous System*.

[30] Li S, Yu M, Li H, Zhang H, Jiang Y (2012). IL-17 and IL-22 in cerebrospinal fluid and plasma are elevated in Guillain-Barré syndrome. *Mediators of Inflammation*.

[31] Liang S-L, Wang W-Z, Huang S, Wang X-K, Zhang S, Wu Y (2012). Th17 helper cell and T-cell immunoglobulin and mucin domain 3 involvement in GuillainBarré syndrome. *Immunopharmacology and Immunotoxicology*.

[32] Pelidou S-H, Zou L-P, Deretzi G, Oniding C, Mix E, Zhu J (2000). Enhancement of acute phase and inhibition of chronic phase of experimental autoimmune neuritis in Lewis rats by intranasal administration of recombinant mouse interleukin 17: potential immunoregulatory role. *Experimental Neurology*.

[33] Shen F, Gaffen SL (2008). Structure-function relationships in the IL-17 receptor: implications for signal transduction and therapy. *Cytokine*.

[34] Zepp J, Wu L, Li X (2011). IL-17 receptor signaling and T helper 17-mediated autoimmune demyelinating disease. *Trends in Immunology*.

[35] Li C, Zhao P, Sun X, Che Y, Jiang Y (2013). Elevated levels of cerebrospinal fluid and plasma interleukin-37 in patients with guillain-barré syndrome. *Mediators of Inflammation*.

[36] McNamee EN, Masterson JC, Jedlicka P (2011). Interleukin 37 expression protects mice from colitis. *Proceedings of the National Academy of Sciences of the United States of America*.

[37] Sakai N, van Sweringen HL, Belizaire RM (2012). Interleukin-37 reduces liver inflammatory injury via effects on hepatocytes and non-parenchymal cells. *Journal of Gastroenterology and Hepatology*.

[38] Kebir H, Kreymborg K, Ifergan I (2007). Human TH17 lymphocytes promote blood-brain barrier disruption and central nervous system inflammation. *Nature Medicine*.

[39] Akashi-Takamura S, Miyake K (2006). Toll-like receptors (TLRs) and immune disorders. *Journal of Infection and Chemotherapy*.

[40] Gries M, Davies L, Liu Y (2012). Response of Toll-like receptors in experimental Guillain-Barré syndrome: a kinetic analysis. *Neuroscience Letters*.

[41] Kuijf ML, Samsom JN, van Rijs W (2010). TLR4-mediated sensing of *Campylobacter jejuni* by dendritic cells is determined by sialylation. *The Journal of Immunology*.

[42] Huizinga R, van Rijs W, Bajramovic JJ (2013). Sialylation of *Campylobacter jejuni* endotoxin promotes dendritic cell-mediated B cell responses through CD14-dependent production of IFN-*β* and TNF-α. *The Journal of Immunology*.

[43] Mercier BC, Cottalorda A, Coupet C-A, Marvel J, Bonnefoy-Bérard N (2009). TLR2 engagement on CD8 T cells enables generation of functional memory cells in response to a suboptimal TCR signal. *Journal of Immunology*.

[44] Zhang ZY, Schluesener HJ (2009). Toll-like receptor-2, CD14 and heat-shock protein 70 in inflammatory lesions of rat experimental autoimmune neuritis. *Neuroscience*.

[45] Laurà M, Mazzeo A, Aguennouz M (2006). Immunolocalization and activation of nuclear factor-*κ*B in the sciatic nerves of rats with experimental autoimmune neuritis. *Journal of Neuroimmunology*.

[46] Wang YZ, Liang QH, Ramkalawan H (2011). Expression of toll-like receptors 2, 4 and 9 in patients with guillain-barré syndrome. *NeuroImmunoModulation*.

[47] Schumann RR, Tapping RI (2007). Genomic variants of TLR1—It takes (TLR-)two to tango. *European Journal of Immunology*.

[48] Müller M, Stenner M, Wacker K, Ringelstein EB, Hickey WF, Kiefer R (2006). Contribution of resident endoneurial macrophages to the local cellular response in experimental autoimmune neuritis. *Journal of Neuropathology and Experimental Neurology*.

[49] Beaino W, Trifilieff E (2010). Thiopalmitoylated peptides from the peripheral nervous system myelin P0 protein: synthesis, characterization, and neuritogenic properties. *Bioconjugate Chemistry*.

[50] Deng Y-N, Zhou W-B (2007). Expression of TLR4 and TLR9 mRNA in Lewis rats with experimental allergic neuritis. *NeuroImmunoModulation*.

[51] Marta M, Andersson Å, Isaksson M, Kämpe O, Lobell A (2008). Unexpected regulatory roles of TLR4 and TLR9 in experimental autoimmune encephalomyelitis. *European Journal of Immunology*.

[52] Pepper M, Dzierszinski F, Wilson E (2008). Plasmacytoid dendritic cells are activated by Toxoplasma gondii to present antigen and produce cytokines. *Journal of Immunology*.

[53] Lancioni CL, Li Q, Thomas JJ (2011). *Mycobacterium tuberculosis* lipoproteins directly regulate human memory CD4^+^ T cell activation via toll-like receptors 1 and 2. *Infection and Immunity*.

[54] Geleijns K, Jacobs BC, van Rijs W, Tio-Gillen AP, Laman JD, van Doorn PA (2004). Functional polymorphisms in LPS receptors CD14 and TLR4 are not associated with disease susceptibility or *Campylobacter jejuni* infection in Guillain-Barré patients. *Journal of Neuroimmunology*.

[55] Koga M, Yuki N, Kashiwase K, Tadokoro K, Juji T, Hirata K (1998). Guillain-Barre and Fisher's syndromes subsequent to *Campylobacter jejuni* enteritis are associated with HLA-B54 and Cw1 independent of anti-ganglioside antibodies. *Journal of Neuroimmunology*.

[56] Geleijns K, Schreuder GMT, Jacobs BC (2005). HLA class II alleles are not a general susceptibility factor in Guillain-barré syndrome. *Neurology*.

[57] Ma JJ, Nishimura M, Mine H (1998). HLA and T-cell receptor gene polymorphisms in Guillain-Barre syndrome. *Neurology*.

[58] Prasad KN, Nyati KK, Verma A, Rizwan A, Paliwal VK (2010). Tumor necrosis factor-α polymorphisms and expression in Guillain-Barré syndrome. *Human Immunology*.

[59] Myhr K-M, Vågnes KS, Marøy TH, Aarseth JH, Nyland HI, Vedeler CA (2003). Interleukin-10 promoter polymorphisms in patients with Guillain-Barré syndrome. *Journal of Neuroimmunology*.

[60] Arbour NC, Lorenz E, Schutte BC (2000). TLR4 mutations are associated with endotoxin hyporesponsiveness in humans. *Nature Genetics*.

[61] Maeda S, Akanuma M, Mitsuno Y (2001). Distinct mechanism of Helicobacter pylori -mediated NF-*κ* B activation between gastric cancer cells and monocytic cells. *Journal of Biological Chemistry*.

[62] Rock FL, Hardiman G, Timans JC, Kastelein RA, Bazan JF (1998). A family of human receptors structurally related to Drosophila Toll. *Proceedings of the National Academy of Sciences of the United States of America*.

[63] Leadbetter EA, Rifkin IR, Hohlbaum AM, Beaudette BC, Shlomchik MJ, Marshak-Rothstein A (2002). Chromatin-IgG complexes activate B cells by dual engagement of IgM and Toll-like receptors. *Nature*.

[64] Nyati KK, Prasad KN, Verma A (2010). Association of TLR4 Asp299Gly and Thr399Ile polymorphisms with Guillain-barré syndrome in Northern Indian population. *Journal of Neuroimmunology*.

[65] Caporale CM, Papola F, Fioroni MA (2006). Susceptibility to Guillain-Barré syndrome is associated to polymorphisms of CD1 genes. *Journal of Neuroimmunology*.

[66] Geleijns K, Roos A, Houwing-Duistermaat JJ (2006). Mannose-binding lectin contributes to the severity of Guillain-Barré syndrome. *Journal of Immunology*.

[67] Sinha S, Prasad KN, Jain D, Nyati KK, Pradhan S, Agrawal S (2010). Immunoglobulin IgG Fc-receptor polymorphisms and HLA class II molecules in Guillain-Barré syndrome. *Acta Neurologica Scandinavica*.

[68] van Sorge NM, van Der Pol W-L, Jansen MD (2005). Severity of Guillain-Barré syndrome is associated with Fc*γ* receptor III polymorphisms. *Journal of Neuroimmunology*.

[69] Uncini A, Notturno F, Pace M, Caporale CM (2011). Polymorphism of CD1 and SH2D2A genes in inflammatory neuropathies. *Journal of the Peripheral Nervous System*.

[70] Pandey JP, Vedeler CA (2003). Immunoglobulin KM genes in Guillain-Barré syndrome. *Neurogenetics*.

[71] Dekker MJHJ, van Den Akker ELT, Koper JW (2009). Effect of glucocorticoid receptor gene polymorphisms in Guillain-Barré syndrome. *Journal of the Peripheral Nervous System*.

[72] Sharshar T, Durand M-C, Lefaucheur J-P (2002). MMP-9 correlates with electrophysiologic abnormalities in Guillain-Barré syndrome. *Neurology*.

[73] Kuwabara S (2004). Guillain-Barré syndrome: epidemiology, pathophysiology and management. *Drugs*.

[74] Vucic S, Kiernan MC, Cornblath DR (2009). Guillain-Barré syndrome: an update. *Journal of Clinical Neuroscience*.

[75] Sharief MK, Ingram DA, Swash M, Thompson EJ (1999). I.v. immunoglobulin reduces circulating proinflammatory cytokines in Guillain-Barré syndrome. *Neurology*.

[76] van der Meche FG, Schmitz PI (1992). A randomized trial comparing intravenous immune globulin and plasma exchange in Guillain-Barré syndrome. Dutch Guillain-Barré Study Group. *The New England Journal of Medicine*.

[77] Thornton CA, Ballow M (1993). Safety of intravenous immunoglobulin. *Archives of Neurology*.

[78] Thornton CA, Griggs RC (1994). Plasma exchange and intravenous immunoglobulin treatment of neuromuscular disease. *Annals of Neurology*.

[79] Raphaël J-C, Chevret S, Chastang C, Jars-Guincestre M-C (1997). Appropriate number of plasma exchanges in Guillain-Barré syndrome. The French Cooperative Group on Plasma Exchange in Guillain-Barré Syndrome. *Annals of Neurology*.

[80] Vriesendorp FJ, Dmytrenko GS, Dietrich T, Koski CL (1993). Anti-peripheral nerve myelin antibodies and terminal activation products of complement in serum of patients with acute brachial plexus neuropathy. *Archives of Neurology*.

[81] Hughes RA, Swan AV, Raphaël J-C, Annane D, van Koningsveld R, van Doorn PA (2007). Immunotherapy for Guillain-Barré syndrome: a systematic review. *Brain*.

[82] Hughes RA, Swan AV, van Doorn PA (2012). Intravenous immunoglobulin for Guillain-Barré syndrome. *Cochrane Database System Review*.

[83] Hughes RA, Newsom-Davis JM, Perkin GD, Pierce JM (1978). Controlled trial of prednisolone in acute polyneuropathy. *The Lancet*.

[84] Singh NK, Gupta A (1996). Do corticosteriods influence the disease course or mortality in Guillain-Barre' syndrome?. *The Journal of Association of Physicians of India*.

